# Laparoscopy and Peritoneal Biopsy in the Diagnosis of Peritoneal Tuberculosis: A Case Report

**DOI:** 10.1002/ccr3.73493

**Published:** 2026-09-09

**Authors:** Sri Ram Charan Gundapaneni, Shravani Divity, Amulya Varshini Banka, Rithwik Goud Burri, Neha Narayan, Yethindra Vityala

**Affiliations:** ^1^ Department of General Medicine Rangaraya Medical College Kakinada India; ^2^ Department of General Medicine Government Medical College Mahabubnagar India; ^3^ Department of Internal Medicine, Sri Laxmi Multi Speciality Hospital Laparoscopic and Research Centre Hyderabad Telangana India; ^4^ Department of General Medicine Maheshwara Medical College and Hospital Hyderabad India; ^5^ Department of General Medicine SVS Medical College Mahabubnagar India; ^6^ Department of Pathology International Higher School of Medicine Bishkek Kyrgyzstan

**Keywords:** anti‐tuberculosis treatment, ascites, biopsy, laparoscopy, peritoneal tuberculosis, pulmonary tuberculosis

## Abstract

A 48‐year‐old male was initially diagnosed with pulmonary tuberculosis (TB) based on presenting symptoms, abdominal complications, and systemic symptoms, despite anti‐TB treatment. Diagnostic laparoscopy and histopathological examination of biopsy samples confirmed peritoneal TB, leading to an adjusted anti‐TB regimen and significant clinical improvement over six months.

## Key Clinical Message

This case highlights the difficulties in peritoneal tuberculosis diagnosis, the value of laparoscopy, and the importance of promptly considering extrapulmonary tuberculosis in high‐risk patients. Combining diagnostic and therapeutic approaches can enhance outcomes, especially in resource‐constrained environments.

## 1 Introduction

Peritoneal tuberculosis (PTB) is a rare form of extrapulmonary manifestation of TB that accounts for 0.5% of new cases and 11% of extrapulmonary forms [1]. TB can spread to the peritoneum through various means, such as the bloodstream from pulmonary TB or the lymphatic system after ingesting contaminated sputum, direct extension from adjacent infected regions via hematogenous or lymphatic dissemination, or progression of nearby foci of infection [2, 3].

PTB presents with vague symptoms, often resulting from the reactivation of peritoneal sites [4, 5]. In the United States, extrapulmonary TB represents 18% of tuberculosis cases, with PTB comprising 6.2% [6, 7]. Risk factors for developing peritonitis include liver cirrhosis, diabetes mellitus, Human Immunodeficiency Virus (HIV), corticosteroid use, malnutrition, and end‐stage renal disease in patients undergoing continuous ambulatory peritoneal dialysis [8].

PTB can occur due to the spread of a nearby tuberculous focus, reactivation of dormant *Mycobacterium (M.) TB* in the mesenteric lymph nodes, or an abdominal focus that has been latent for years [9]. Certain factors can increase the likelihood of *M. TB* spread, such as chronic liver disease, oncological processes, immunosuppression, and the use of cytostatics. Patients with rheumatological diseases, autoimmune diseases, and solid organ transplants treated with immunosuppressive drugs are also at risk, as are those with HIV/AIDS, pregnancy, and childbirth [10, 11].

The clinical presentation of ascites syndrome can resemble that of cirrhotic patients, making an accurate diagnosis crucial. Imaging studies, such as chest X‐ray, abdominal ultrasound, computed tomography (CT), and echocardiogram, can reveal the presence of free fluid or septate fluid in the peritoneal cavity and mesenteric and omentum involvement, which may be accompanied by increased density or solid masses [1, 2, 12, 13].

Laboratory tests, including clinical examination of peritoneal fluid, determination of adenosine deaminase, and molecular biology techniques (polymerase chain reaction), can provide valuable diagnostic information [9]. Although imaging advances have decreased the usefulness of laparoscopy in other entities, it remains an essential tool in the diagnosis of PTB, as it allows for the collection of biopsy samples to differentiate between peritoneal carcinosis, mesothelioma, and bacterial peritonitis [14].

Patients with PTB should receive anti‐TB treatment according to the pulmonary TB protocol [15, 16]. When the diagnosis is inconclusive but suspicion is strong based on symptoms, epidemiological context, and increased ascitic fluid adenosine deaminase (ADA) or matching histological findings, empiric anti‐TB therapy is justified [17]. Fever subsides within weeks, and patients with ascites or tuberculous enteritis recover within weeks. However, healing may cause scar‐related strictures, requiring surgery for perforation, abscesses, fistulas, bleeding, or severe obstruction. Among 106 patients with intestinal TB strictures, only 25% had resolution after treatment [18].

In this case, the coexistence of PTB and pulmonary TB serves as a reminder of the importance of considering active TB infections in the differential diagnosis.

## 2 Case Presentation

### 2.1 Past History and Initial Presentation

A 48‐year‐old man with a history of tobacco and alcohol consumption presented with a fever and significant night sweats. Over five days, he developed a productive cough with light‐red sputum.

### 2.2 Physical Examination

Initial physical examination was unremarkable.

### 2.3 Diagnostic Evaluations and Methods

The diagnostic process included clinical evaluation, laboratory tests, microbiological analyses, imaging, ascitic fluid analysis, and histopathological examination. The clinical indicators included fever, night sweats, productive cough, abdominal pain, swelling, nausea, vomiting, and lower limb edema. Laboratory tests included erythrocyte sedimentation rate, complete blood count, liver function tests (alanine transaminase, aspartate transaminase, and gamma‐glutamyl transferase), serum total protein, albumin, and serology for HIV, hepatitis B, and hepatitis C. Microbiological assessments involved sputum acid‐fast bacilli microscopy and GeneXpert MTB/RIF testing for *M. TB* and rifampicin resistance testing. Radiological evaluations included chest radiography, abdominal ultrasonography, and contrast‐enhanced CT to assess lung disease, ascites, peritoneal thickening, and omental involvement. Diagnostic paracentesis was used to analyze the ascitic fluid, and laparoscopy with targeted biopsy provided a definitive diagnosis. Histopathological examination revealed granulomatous inflammation, epithelioid histiocytes, and Langhans giant cells, while culture confirmed *M. TB*, distinguishing PTB from malignancy and chronic liver disease.

### 2.4 Initial Diagnostic Workup

Tests revealed elevated markers such as erythrocyte sedimentation rate, 18 mm/h; alanine transaminase, 39 U/L; aspartate transaminase, 88 U/L. Decreased levels of total protein (61 g/L) and cholesterol (2.9 mmol/L). Sputum analysis for acid‐fast bacilli revealed positive results. Chest radiography revealed a normal cardiothoracic ratio without pleuropulmonary abnormalities. Mantoux test results were hyperergic at 18 mm. Pulmonary tuberculosis was confirmed, leading to the initiation of antituberculosis treatment.

### 2.5 Clinical Course and Treatment Adjustments

The initial phase included daily doses of isoniazid (5 mg/kg), rifampicin (10 mg/kg), pyrazinamide (20 mg/kg), and ethambutol (60 doses of 15 mg/kg). Despite the improvement, the patient continued to experience occasional cough and blood‐tinged sputum. Follow‐up examination revealed abdominal discomfort, bloating, nausea, vomiting, and leg swelling. Repeated sputum analysis for BAAR was positive, indicating treatment failure and necessitating a hospital referral.

### 2.6 Hospital Admission and Clinical Examination

Upon hospital admission, examination revealed a slightly diminished vesicular murmur was more pronounced in the upper right lobe, with fine crackles. Abdominal examination revealed a symmetrical, tender abdomen; no masses or organ enlargement; bowel sounds present. Positive Tarral sign. Subcutaneous tissue revealed cold, soft, and compressible edema in both lower limbs.

### 2.7 Advanced Diagnostic Findings

**FIGURE 1:**
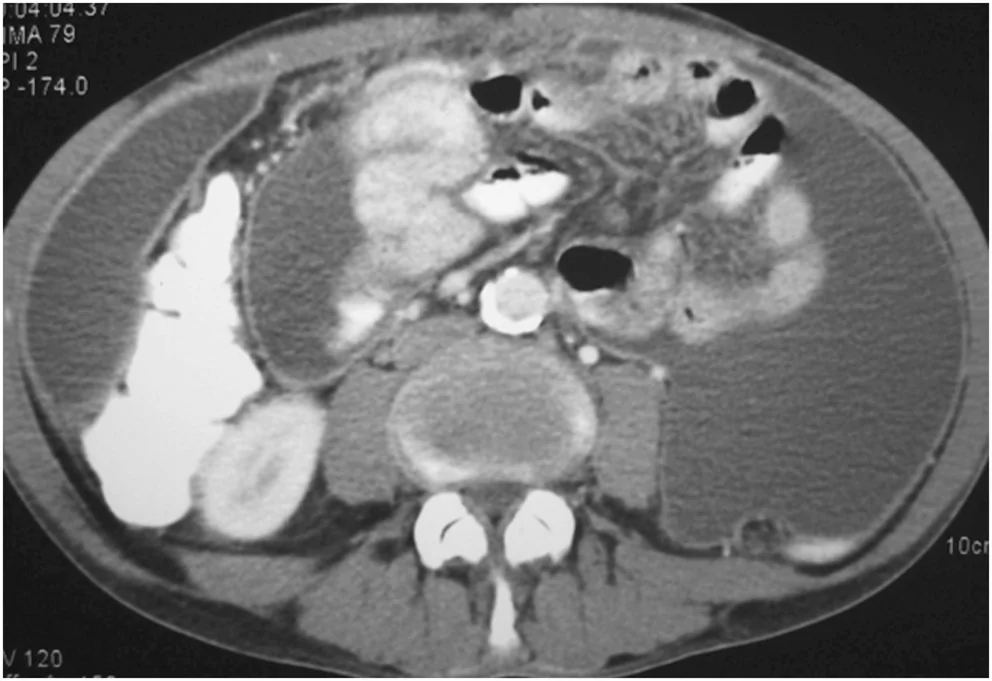
Abdominal computed tomography showed septate ascitic fluid in various locations, with significant thickening of the parietal peritoneum.

Platelets: 145 × 10^9/L (below normal). ESR: 25 mm/h (slightly elevated). Blood chemistry revealed elevated alanine transaminase (77 U/L), aspartate transaminase (98 U/L), and gamma‐glutamyl transferase (62 U/L) levels and decreased total protein (49 g/L) and albumin (21 g/L) levels. HIV antibody test result was negative. Hepatitis B surface antigen or hepatitis C virus antibody was not detected. Negative serology GENE (X‐Pert) MTB/RIF (real‐time PCR for *Mycobacterium tuberculosis* and rifampicin‐resistance mutations) was positive, indicating monoresistance to isoniazid or rifampicin. Abdominal ultrasound showed a normal‐sized liver with an altered echostructure, uneven surface, predominance of fine nodules, free fluid in the abdominal cavity, normal‐sized spleen, and thickened peritoneum. Abdominal computed tomography depicted septal ascites with significant peritoneal and omental thickening, and a normal liver and spleen (Figure 1).

### 2.8 Laparoscopy and Biopsy Findings

**FIGURE 2:**
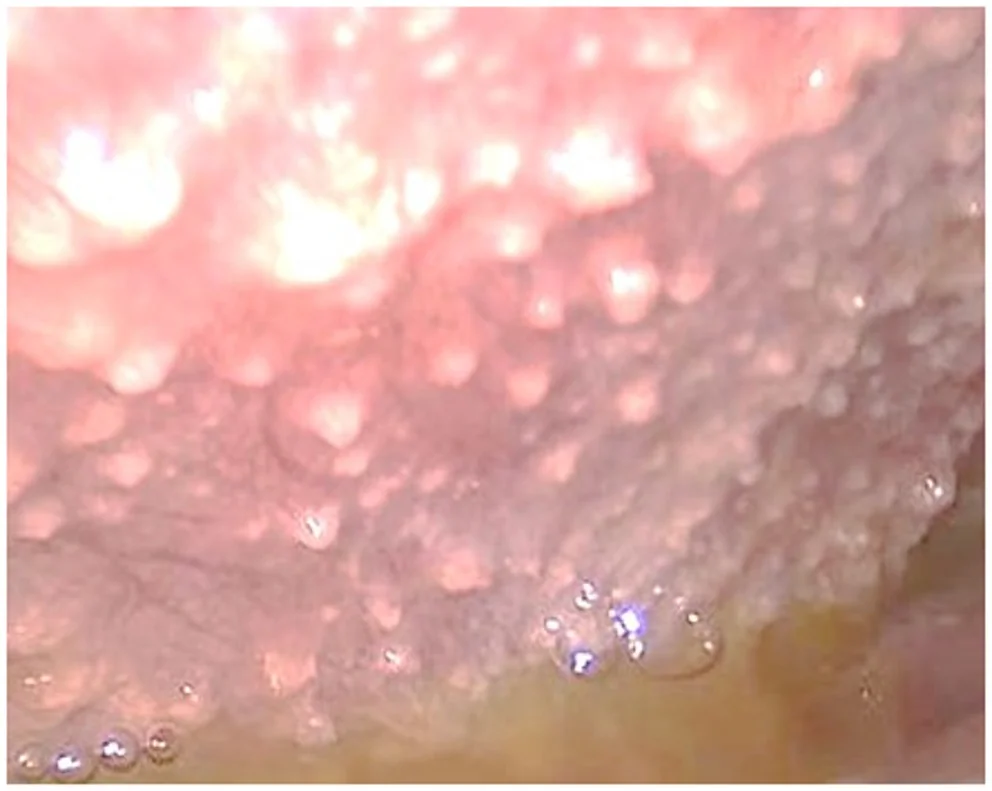
Laparoscopic image showing tuberculate formations on the peritoneum and serous exudate.

**FIGURE 3:**
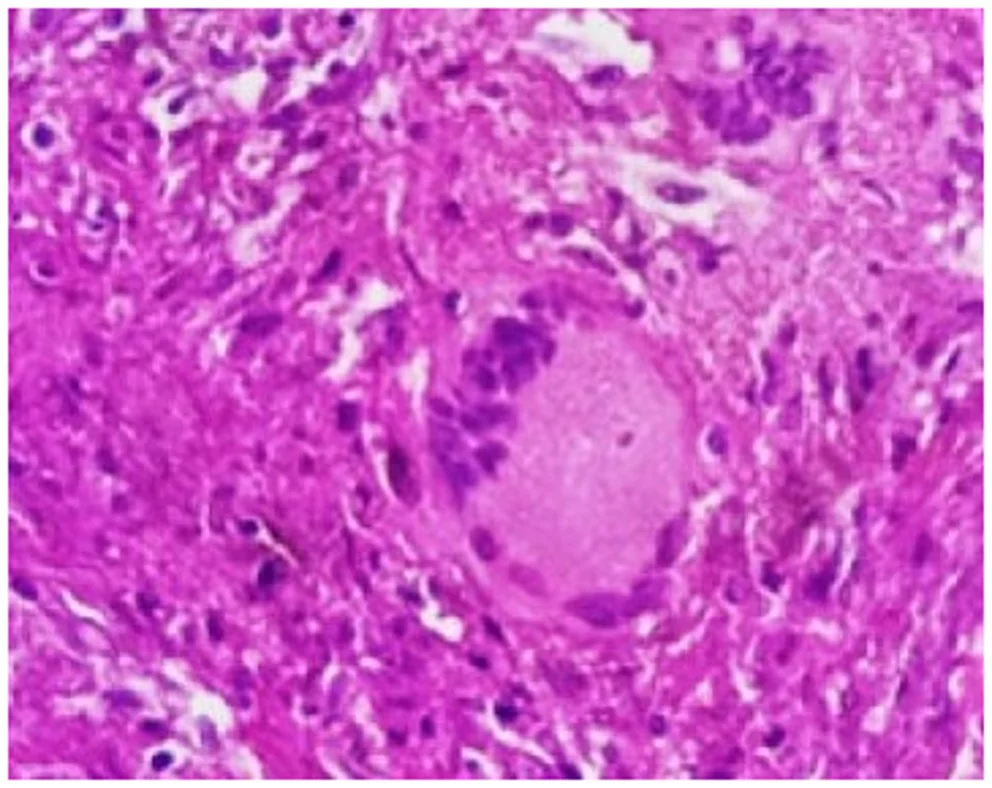
Hematoxylin and eosin staining demonstrating Langhans giant cells and epithelioid cell granulomas with central necrosis.

After evaluating the epidemiological factors, physical examination findings, paraclinical test results, and hypotheses, diagnostic laparoscopy with biopsy was performed. Preceding the procedure, 1,000 mL of cloudy yellow fluid was extracted and analyzed chemically, bacteriologically, and cytologically, revealing 660 cells/μL, primarily lymphocytes. Laparoscopy revealed a nodular liver surface with irregular edges and white, rice‐like clumps on the peritoneum, accompanied by serous exudate (Figure 2). Histological examination of the tissue sample showed histiocytes and Langhans giant cells that formed granulomas that lacked malignant characteristics (Figure 3), which were identified as tuberculous peritonitis.

### 2.9 Definitive Diagnosis and Initiation of Treatment

Mycobacterial cultures subsequently confirmed the *M. TB* infection. An 18‐month treatment protocol consisting of ethambutol (20 mg/kg/day), moxifloxacin (1 mg/kg/day), terizidone (15 mg/kg/day), and capreomycin (15 mg/kg/day) was initiated.

### 2.10 Follow‐Up

The patient showed a satisfactory improvement over six months.

## 3 Discussion

PTB frequently causes gradual abdominal swelling due to ascites and discomfort, along with general symptoms like fever, night sweats, and weight loss, which can develop slowly over months before medical intervention [19, 20, 21]. Diagnosing PTB is challenging, especially in cirrhotic patients, as symptoms are non‐specific and resemble other abdominal conditions, while typical TB symptoms are often absent without concurrent active pulmonary TB [19].

Hepatorenal syndrome was the immediate cause of death, with peritoneal carcinosis as the underlying cause, but pathological examination revealed widespread sepsis due to active miliary TB spreading to the peritoneum, underscoring the complexities in diagnosing PTB [6, 22, 23, 24]. PTB prevalence reflects inadequacies in pulmonary TB diagnosis or correlates with poverty rates, as indicated by a country's GDP and healthcare spending, with lower incidence in developed nations [25].

The patient's case indicated chronic liver disease, diagnosed as alcoholic liver cirrhosis based on heavy alcohol use and negative hepatitis B and C tests, which worsened because of the liver‐toxic effects of anti‐TB medications. While abdominal ultrasound and CT scans can aid in diagnosing pulmonary TB, results may be inconclusive [26], and one study found laparoscopy provided additional information in 31% of patients compared to imaging [27].

Laparoscopy and laparotomy effectively confirmed TB without affecting treatment or causing complications [24, 28, 29]. Invasive diagnostic procedures may be necessary to diagnose PTB, which can be difficult to identify. The most reliable method for diagnosing PTB is peritonoscopy with peritoneal biopsy, which shows three patterns: A thickened peritoneum with yellow‐white tubercles, thickened peritoneum without tubercles, or significant peritoneal thickening with dense adhesions [24, 29]. Acid‐fast staining of peritoneal biopsy specimens is positive in 50%–63% of cases, while culture is positive in approximately 70% of cases [30, 31, 32]. Histopathological examination revealed caseating granulomas in 70%–95% of the patients, and PCR of the tissue was positive in 25%–70% of the cases [20, 21, 23, 29, 30, 31, 32].

Ascitic fluid analysis is commonly used initially, but its effectiveness is limited to certain conditions. Smear microscopy for acid‐fast bacilli has low sensitivity due to the paucibacillary nature of the disease, and mycobacterial cultures may take weeks. Ascitic fluid ADA is a valuable biomarker, and studies have shown sensitivities and specificities above 90% at the appropriate thresholds. However, interpret ADA alongside clinical, radiological, and microbiological findings, as elevated levels occur in other inflammatory conditions [33, 34, 35].

The microbiological culture process can take up to eight weeks, delaying diagnosis and treatment. The UK National Institute for Clinical Excellence recommends initiating treatment for extrapulmonary TB if histology and clinical features indicate pulmonary TB, without waiting for culture results [27].

Molecular diagnostics have improved the assessment of suspected extrapulmonary TB. GeneXpert MTB/RIF and similar nucleic acid amplification tests rapidly detect *M. TB* and drug resistance. However, the sensitivity of extrapulmonary samples varies with bacterial load and specimen quality; a negative molecular test result should not exclude the diagnosis when clinical suspicion remains high. Here, combining microbiological, radiological, laparoscopic, and histopathological findings was crucial for diagnostic certainty and treatment decisions [36, 37].

Excluding PTB requires consideration of immunological and socio‐epidemiological risk factors, including travel history, homelessness, imprisonment, contact with ill individuals, and drug use. Traditional symptoms such as fever, weight loss, and night sweats may not always be present. Diagnostic procedures include Ziehl‐Neelsen staining and culture, peritoneal biopsy, interferon‐γ release assays or tuberculin skin tests, ADA measurement in ascitic fluid, and molecular methods. Clinical, radiographic, and cytological responses to treatment also help confirm the diagnosis.

Diagnostic laparoscopy is one of the most effective tools for unexplained ascites and suspected PTB. Direct visualization identified features such as whitish tubercles, peritoneal thickening, adhesions, hyperemia, and omental involvement. More importantly, laparoscopy enables targeted tissue sampling, increasing the diagnostic yield over blind biopsies. Previous studies report diagnostic accuracies exceeding 85%–95% when combined with histopathological examination and microbiological culture, confirming its status as the gold standard in complex cases [38, 39].

Laparoscopy and peritoneal biopsy provided a definitive diagnosis of PTB in this patient, demonstrating their utility in ambiguous cases by facilitating histopathological confirmation and differential diagnosis. Histological examination revealed caseating granulomas, a hallmark of TB, and microbiological tests confirmed *M. TB*.

This study has some limitations. As a single‐patient case report, the findings may not apply to all patients with PTB. Although laparoscopy, histopathology, and culture confirmed the diagnosis, biomarkers such as ascitic fluid ADA, interferon‐gamma tests, and molecular methods were not used and might have provided insight. The lack of follow‐up beyond six months limits the assessment of treatment durability, recurrence, and long‐term complications. Chronic liver disease may influence the presentation and treatment, complicating interpretation.

Future studies should focus on improving the non‐invasive diagnosis of PTB in resource‐constrained settings. Rapid molecular tests, biomarker frameworks, and artificial intelligence‐assisted imaging may facilitate earlier diagnosis. Prospective multicenter studies are required to evaluate new biomarkers, standardize protocols, and examine treatment response, drug resistance, and long‐term outcomes.

This case highlights the coexistence of pulmonary and PTB; persistent abdominal symptoms despite anti‐TB treatment should prompt evaluation for treatment failure, drug resistance, or extrapulmonary spread. This highlights the liver toxicity associated with prolonged anti‐TB treatment, particularly in patients with pre‐existing liver issues. The integration of imaging, molecular diagnostics, and minimally invasive procedures is crucial for diagnosis and treatment, improving outcomes.

This case is noteworthy because the diagnosis of PTB was established in a patient who was already receiving treatment for Pulmonary TB and subsequently developed persistent abdominal manifestations suggestive of treatment failure or an alternative pathology. The report highlights the diagnostic value of laparoscopic visualization combined with peritoneal biopsy in differentiating PTB from cirrhosis‐related ascites, peritoneal carcinomatosis, and other causes of chronic peritoneal inflammation. Furthermore, it demonstrates the importance of maintaining a high index of suspicion for extrapulmonary involvement despite ongoing anti‐TB therapy.

## 4 Conclusions

PTB is a challenging extrapulmonary TB form because its vague symptoms can mimic those of infectious, inflammatory, and malignant diseases. This case underscores the need for a high level of suspicion in patients with unexplained ascites, systemic symptoms, and a history of or risk factors for TB, especially in endemic areas. Although laboratory tests and imaging may help, they may not confirm the diagnosis.

Diagnostic laparoscopy with targeted peritoneal biopsy was crucial because it enabled direct visualization of typical lesions and histopathological and microbiological confirmation of diagnosis. These findings support minimally invasive tissue diagnosis when non‐invasive tests are inconclusive and the consideration of extrapulmonary spread during pulmonary TB treatment.

Early recognition and diagnosis allow timely treatment, reduce delays, avoid unnecessary procedures, and improve patient outcomes. Integrated clinical, radiological, microbiological, molecular, and histopathological assessments remain vital for the effective management of this condition in practice across endemic regions, and more precise, accessible, noninvasive tools could improve early detection and treatment, particularly in resource‐constrained high‐burden settings.

## Author Contributions

**Sri Ram Charan Gundapaneni:** conceptualization, investigation, methodology, software, validation, writing – original draft, writing – review and editing. **Shravani Divity:** conceptualization, data curation, formal analysis, methodology, writing – original draft, writing – review and editing. **Amulya Varshini Banka:** data curation, investigation, methodology, validation, writing – original draft, writing – review and editing. **Rithwik Goud Burri:** conceptualization, data curation, methodology, supervision, visualization, writing – original draft, writing – review and editing. **Neha Narayan:** conceptualization, investigation, methodology, resources, writing – original draft, writing – review and editing. **Yethindra Vityala:** conceptualization, data curation, formal analysis, investigation, methodology, software, validation, writing – original draft, writing – review and editing.

## Funding

The authors have nothing to report.

## Consent

Written informed consent was obtained from the patient to publish this report in accordance with the journal's patient consent policy.

## Conflicts of Interest

The authors declare no conflicts of interest.

## Data Availability

Data are available from the corresponding author upon reasonable request.
